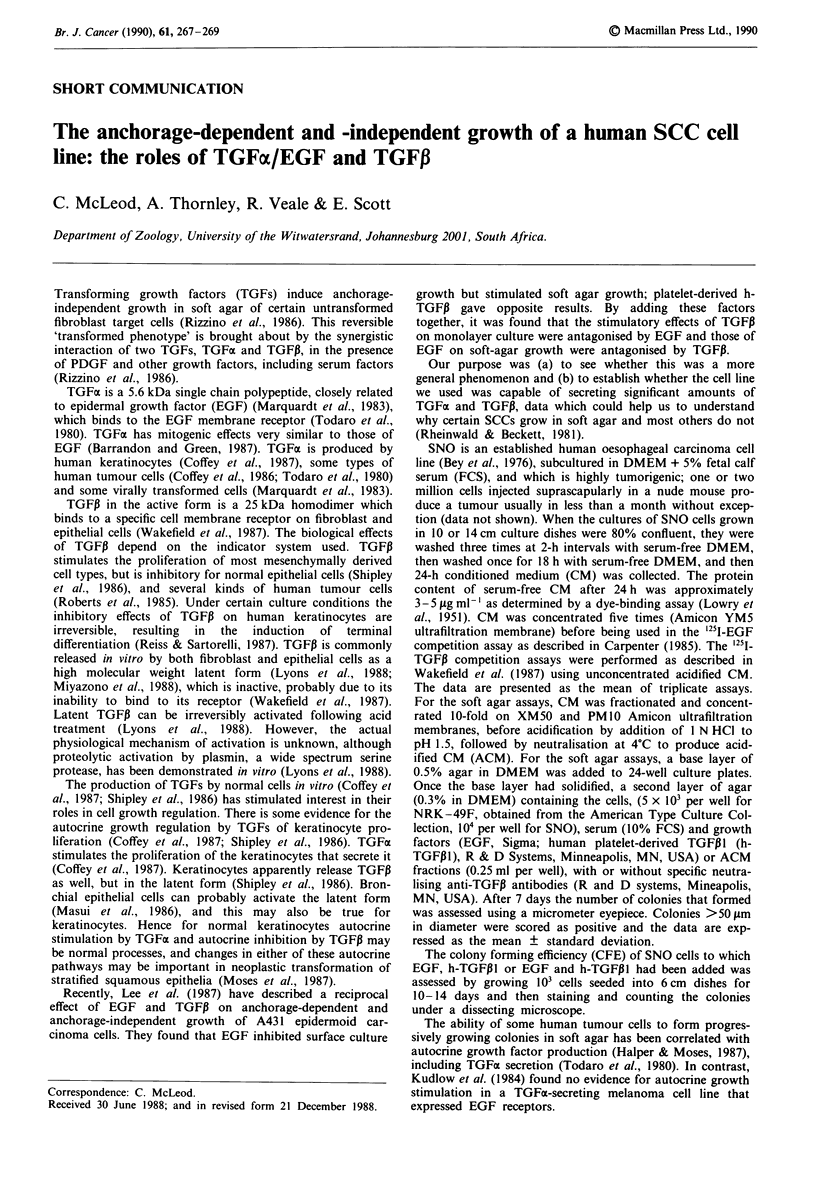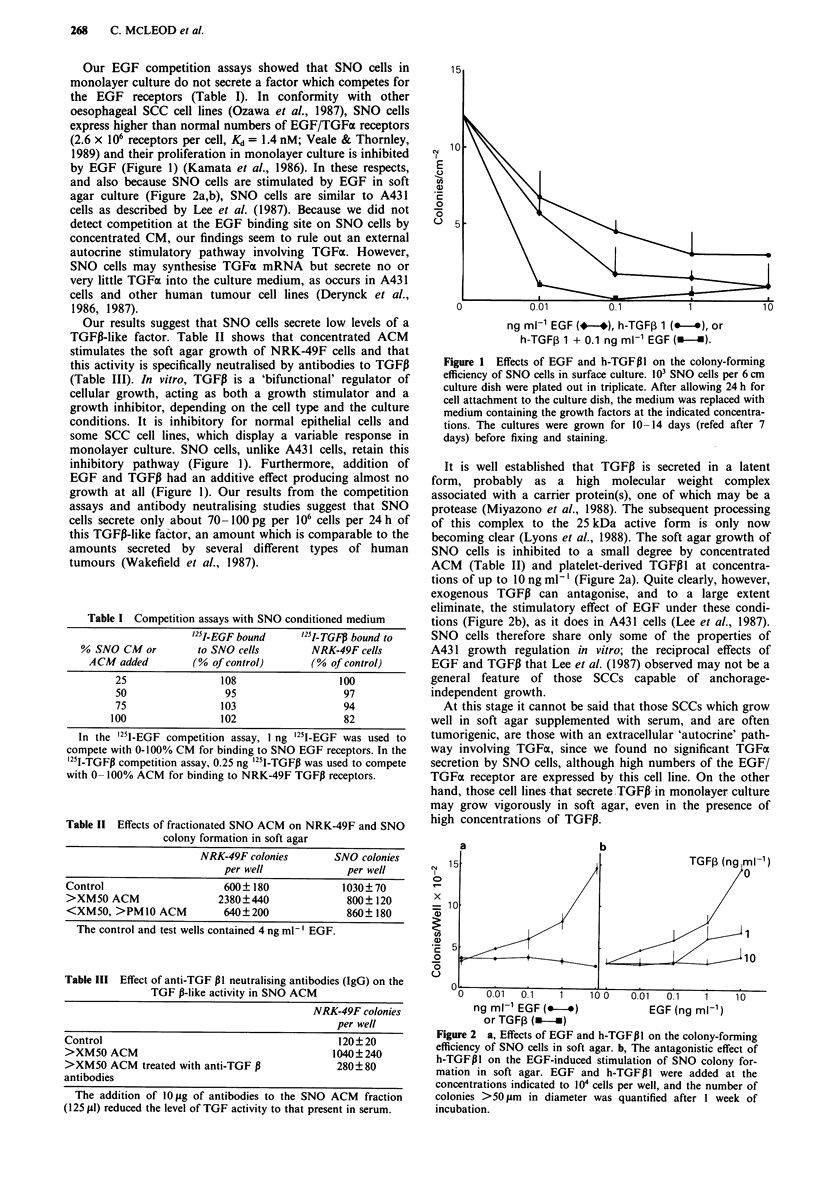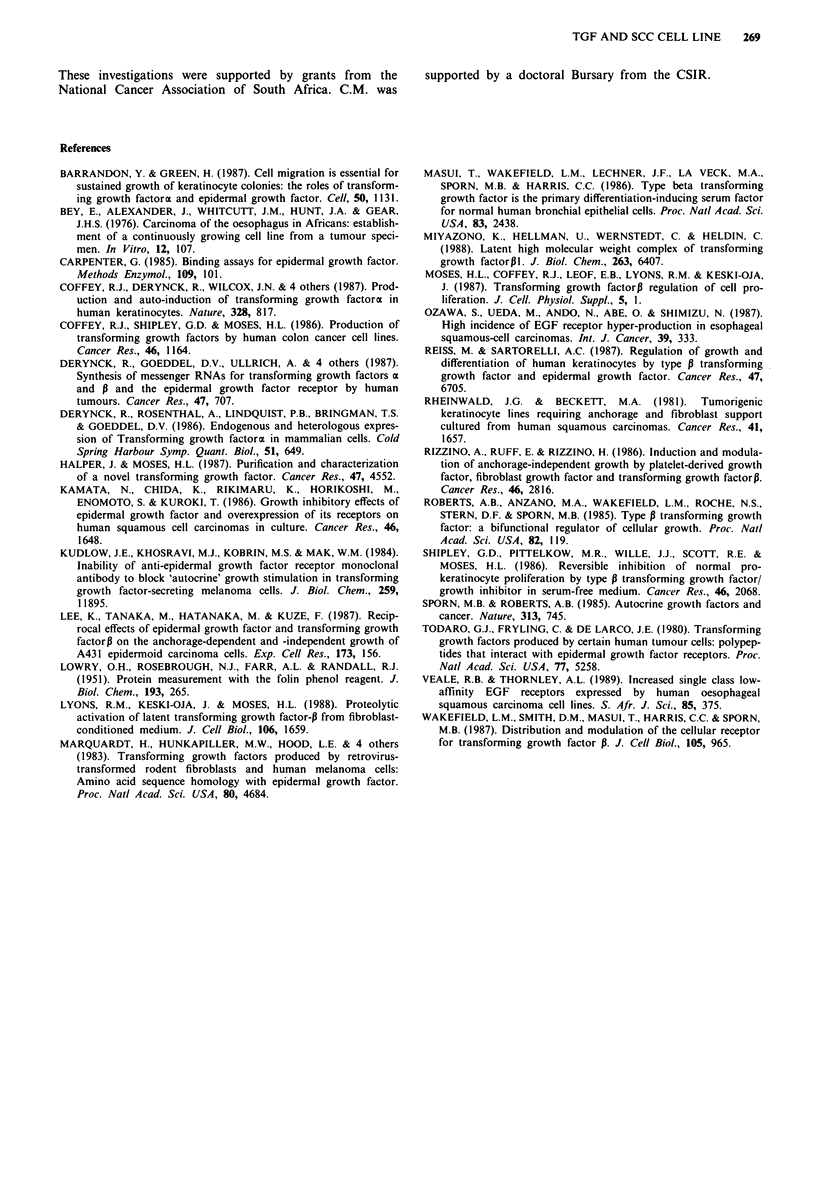# The anchorage-dependent and -independent growth of a human SCC cell line: the roles of TGF alpha/EGF and TGF beta.

**DOI:** 10.1038/bjc.1990.49

**Published:** 1990-02

**Authors:** C. McLeod, A. Thornley, R. Veale, E. Scott

**Affiliations:** Department of Zoology, University of the Witwatersrand, Johannesburg, South Africa.


*Br. J. Cancer* (1990), **61**, 267–269 © Macmillan Press Ltd., 1990

SHORT COMMUNICATION

### The anchorage-dependent and -independent growth of a human SCC cell line: the roles of TGFα/EGF and TGFβ

C. McLeod, A. Thornley, R. Veale & E. Scott

*Department of Zoology, University of the Witwatersrand, Johannesburg 2001, South Africa.*

Transforming growth factors (TGFs) induce anchorage-independent growth in soft agar of certain untransformed fibroblast target cells (Rizzino *et al.*, 1986). This reversible 'transformed phenotype' is brought about by the synergistic interaction of two TGFs, TGFα and TGFβ, in the presence of PDGF and other growth factors, including serum factors (Rizzino *et al.*, 1986).

TGFα is a 5.6 kDa single chain polypeptide, closely related to epidermal growth factor (EGF) (Marquardt *et al.*, 1983), which binds to the EGF membrane receptor (Todaro *et al.*, 1980). TGFα has mitogenic effects very similar to those of EGF (Barrandon and Green, 1987). TGFα is produced by human keratinocytes (Coffey *et al.*, 1987), some types of human tumour cells (Coffey *et al.*, 1986; Todaro *et al.*, 1980) and some virally transformed cells (Marquardt *et al.*, 1983).

TGFβ in the active form is a 25 kDa homodimer which binds to a specific cell membrane receptor on fibroblast and epithelial cells (Wakefield *et al.*, 1987). The biological effects of TGFβ depend on the indicator system used. TGFβ stimulates the proliferation of most mesenchymally derived cell types, but is inhibitory for normal epithelial cells (Shipley *et al.*, 1986), and several kinds of human tumour cells (Roberts *et al.*, 1985). Under certain culture conditions the inhibitory effects of TGFβ on human keratinocytes are irreversible, resulting in the induction of terminal differentiation (Reiss & Sartorelli, 1987). TGFβ is commonly released *in vitro* by both fibroblast and epithelial cells as a high molecular weight latent form (Lyons *et al.*, 1988; Miyazono *et al.*, 1988), which is inactive, probably due to its inability to bind to its receptor (Wakefield *et al.*, 1987). Latent TGFβ can be irreversibly activated following acid treatment (Lyons *et al.*, 1988). However, the actual physiological mechanism of activation is unknown, although proteolytic activation by plasmin, a wide spectrum serine protease, has been demonstrated *in vitro* (Lyons *et al.*, 1988).

The production of TGFs by normal cells *in vitro* (Coffey *et al.*, 1987; Shipley *et al.*, 1986) has stimulated interest in their roles in cell growth regulation. There is some evidence for the autocrine growth regulation by TGFs of keratinocyte proliferation (Coffey *et al.*, 1987; Shipley *et al.*, 1986). TGFα stimulates the proliferation of the keratinocytes that secrete it (Coffey *et al.*, 1987). Keratinocytes apparently release TGFβ as well, but in the latent form (Shipley *et al.*, 1986). Bronchial epithelial cells can probably activate the latent form (Masui *et al.*, 1986), and this may also be true for keratinocytes. Hence for normal keratinocytes autocrine stimulation by TGFα and autocrine inhibition by TGFβ may be normal processes, and changes in either of these autocrine pathways may be important in neoplastic transformation of stratified squamous epithelia (Moses *et al.*, 1987).

Recently, Lee *et al.* (1987) have described a reciprocal effect of EGF and TGFβ on anchorage-dependent and anchorage-independent growth of A431 epidermoid carcinoma cells. They found that EGF inhibited surface culture growth but stimulated soft agar growth; platelet-derived h-TGFβ gave opposite results. By adding these factors together, it was found that the stimulatory effects of TGFβ on monolayer culture were antagonised by EGF and those of EGF on soft-agar growth were antagonised by TGFβ.

Our purpose was (a) to see whether this was a more general phenomenon and (b) to establish whether the cell line we used was capable of secreting significant amounts of TGFα and TGFβ, data which could help us to understand why certain SCCs grow in soft agar and most others do not (Rheinwald & Beckett, 1981).

SNO is an established human oesophageal carcinoma cell line (Bey *et al.*, 1976), subcultured in DMEM + 5% fetal calf serum (FCS), and which is highly tumorigenic; one or two million cells injected suprascapularly in a nude mouse produce a tumour usually in less than a month without exception (data not shown). When the cultures of SNO cells grown in 10 or 14 cm culture dishes were 80% confluent, they were washed three times at 2-h intervals with serum-free DMEM, then washed once for 18 h with serum-free DMEM, and then 24-h conditioned medium (CM) was collected. The protein content of serum-free CM after 24 h was approximately 3–5 $\mu g\ ml^{-1}$ as determined by a dye-binding assay (Lowry *et al.*, 1951). CM was concentrated five times (Amicon YM5 ultrafiltration membrane) before being used in the $^{125}$I-EGF competition assay as described in Carpenter (1985). The $^{125}$I-TGFβ competition assays were performed as described in Wakefield *et al.* (1987) using unconcentrated acidified CM. The data are presented as the mean of triplicate assays. For the soft agar assays, CM was fractionated and concentrated 10-fold on XM50 and PM10 Amicon ultrafiltration membranes, before acidification by addition of 1 N HCl to pH 1.5, followed by neutralisation at 4°C to produce acidified CM (ACM). For the soft agar assays, a base layer of 0.5% agar in DMEM was added to 24-well culture plates. Once the base layer had solidified, a second layer of agar (0.3% in DMEM) containing the cells, ($5 \times 10^3$ per well for NRK-49F, obtained from the American Type Culture Collection, $10^4$ per well for SNO), serum (10% FCS) and growth factors (EGF, Sigma; human platelet-derived TGFβ1 (h-TGFβ1), R & D Systems, Minneapolis, MN, USA) or ACM fractions (0.25 ml per well), with or without specific neutralising anti-TGFβ antibodies (R and D systems, Mineapolis, MN, USA). After 7 days the number of colonies that formed was assessed using a micrometer eyepiece. Colonies $>50\ \mu m$ in diameter were scored as positive and the data are expressed as the mean ± standard deviation.

The colony forming efficiency (CFE) of SNO cells to which EGF, h-TGFβ1 or EGF and h-TGFβ1 had been added was assessed by growing $10^3$ cells seeded into 6 cm dishes for 10–14 days and then staining and counting the colonies under a dissecting microscope.

The ability of some human tumour cells to form progressively growing colonies in soft agar has been correlated with autocrine growth factor production (Halper & Moses, 1987), including TGFα secretion (Todaro *et al.*, 1980). In contrast, Kudlow *et al.* (1984) found no evidence for autocrine growth stimulation in a TGFα-secreting melanoma cell line that expressed EGF receptors.

Correspondence: C. McLeod.
Received 30 June 1988; and in revised form 21 December 1988.

Our EGF competition assays showed that SNO cells in monolayer culture do not secrete a factor which competes for the EGF receptors (Table I). In conformity with other oesophageal SCC cell lines (Ozawa et al., 1987), SNO cells express higher than normal numbers of EGF/TGFα receptors (2.6 × $10^6$ receptors per cell, $K_d$ = 1.4 nM; Veale & Thornley, 1989) and their proliferation in monolayer culture is inhibited by EGF (Figure 1) (Kamata et al., 1986). In these respects, and also because SNO cells are stimulated by EGF in soft agar culture (Figure 2a,b), SNO cells are similar to A431 cells as described by Lee et al. (1987). Because we did not detect competition at the EGF binding site on SNO cells by concentrated CM, our findings seem to rule out an external autocrine stimulatory pathway involving TGFα. However, SNO cells may synthesise TGFα mRNA but secrete no or very little TGFα into the culture medium, as occurs in A431 cells and other human tumour cell lines (Derynck et al., 1986, 1987).

Our results suggest that SNO cells secrete low levels of a TGFβ-like factor. Table II shows that concentrated ACM stimulates the soft agar growth of NRK-49F cells and that this activity is specifically neutralised by antibodies to TGFβ (Table III). *In vitro*, TGFβ is a 'bifunctional' regulator of cellular growth, acting as both a growth stimulator and a growth inhibitor, depending on the cell type and the culture conditions. It is inhibitory for normal epithelial cells and some SCC cell lines, which display a variable response in monolayer culture. SNO cells, unlike A431 cells, retain this inhibitory pathway (Figure 1). Furthermore, addition of EGF and TGFβ had an additive effect producing almost no growth at all (Figure 1). Our results from the competition assays and antibody neutralising studies suggest that SNO cells secrete only about 70–100 pg per $10^6$ cells per 24 h of this TGFβ-like factor, an amount which is comparable to the amounts secreted by several different types of human tumours (Wakefield et al., 1987).

**Table I** Competition assays with SNO conditioned medium

| *% SNO CM or ACM added* | *$^{125}$I-EGF bound to SNO cells (% of control)* | *$^{125}$I-TGFβ bound to NRK-49F cells (% of control)* |
|---|---|---|
| 25 | 108 | 100 |
| 50 | 95 | 97 |
| 75 | 103 | 94 |
| 100 | 102 | 82 |

In the $^{125}$I-EGF competition assay, 1 ng $^{125}$I-EGF was used to compete with 0-100% CM for binding to SNO EGF receptors. In the $^{125}$I-TGFβ competition assay, 0.25 ng $^{125}$I-TGFβ was used to compete with 0–100% ACM for binding to NRK-49F TGFβ receptors.

**Table II** Effects of fractionated SNO ACM on NRK-49F and SNO colony formation in soft agar

| | *NRK-49F colonies per well* | *SNO colonies per well* |
|---|---|---|
| Control | 600 ± 180 | 1030 ± 70 |
| >XM50 ACM | 2380 ± 440 | 800 ± 120 |
| <XM50, >PM10 ACM | 640 ± 200 | 860 ± 180 |

The control and test wells contained 4 ng ml$^{-1}$ EGF.

**Table III** Effect of anti-TGF β1 neutralising antibodies (IgG) on the TGF β-like activity in SNO ACM

| | *NRK-49F colonies per well* |
|---|---|
| Control | 120 ± 20 |
| >XM50 ACM | 1040 ± 240 |
| >XM50 ACM treated with anti-TGF β antibodies | 280 ± 80 |

The addition of 10 μg of antibodies to the SNO ACM fraction (125 μl) reduced the level of TGF activity to that present in serum.

**Figure 1** Effects of EGF and h-TGFβ1 on the colony-forming efficiency of SNO cells in surface culture. $10^3$ SNO cells per 6 cm culture dish were plated out in triplicate. After allowing 24 h for cell attachment to the culture dish, the medium was replaced with medium containing the growth factors at the indicated concentrations. The cultures were grown for 10–14 days (refed after 7 days) before fixing and staining.

It is well established that TGFβ is secreted in a latent form, probably as a high molecular weight complex associated with a carrier protein(s), one of which may be a protease (Miyazono et al., 1988). The subsequent processing of this complex to the 25 kDa active form is only now becoming clear (Lyons et al., 1988). The soft agar growth of SNO cells is inhibited to a small degree by concentrated ACM (Table II) and platelet-derived TGFβ1 at concentrations of up to 10 ng ml$^{-1}$ (Figure 2a). Quite clearly, however, exogenous TGFβ can antagonise, and to a large extent eliminate, the stimulatory effect of EGF under these conditions (Figure 2b), as it does in A431 cells (Lee et al., 1987). SNO cells therefore share only some of the properties of A431 growth regulation *in vitro*; the reciprocal effects of EGF and TGFβ that Lee et al. (1987) observed may not be a general feature of those SCCs capable of anchorage-independent growth.

At this stage it cannot be said that those SCCs which grow well in soft agar supplemented with serum, and are often tumorigenic, are those with an extracellular 'autocrine' pathway involving TGFα, since we found no significant TGFα secretion by SNO cells, although high numbers of the EGF/TGFα receptor are expressed by this cell line. On the other hand, those cell lines that secrete TGFβ in monolayer culture may grow vigorously in soft agar, even in the presence of high concentrations of TGFβ.

**Figure 2** a, Effects of EGF and h-TGFβ1 on the colony-forming efficiency of SNO cells in soft agar. b, The antagonistic effect of h-TGFβ1 on the EGF-induced stimulation of SNO colony formation in soft agar. EGF and h-TGFβ1 were added at the concentrations indicated to $10^4$ cells per well, and the number of colonies >50 μm in diameter was quantified after 1 week of incubation.

These investigations were supported by grants from the National Cancer Association of South Africa. C.M. was supported by a doctoral Bursary from the CSIR.

### References

BARRANDON, Y. & GREEN, H. (1987). Cell migration is essential for sustained growth of keratinocyte colonies: the roles of transforming growth factorα and epidermal growth factor. *Cell*, **50**, 1131.

BEY, E., ALEXANDER, J., WHITCUTT, J.M., HUNT, J.A. & GEAR, J.H.S. (1976). Carcinoma of the oesophagus in Africans: establishment of a continuously growing cell line from a tumour specimen. *In Vitro*, **12**, 107.

CARPENTER, G. (1985). Binding assays for epidermal growth factor. *Methods Enzymol.*, **109**, 101.

COFFEY, R.J., DERYNCK, R., WILCOX, J.N. & 4 others (1987). Production and auto-induction of transforming growth factorα in human keratinocytes. *Nature*, **328**, 817.

COFFEY, R.J., SHIPLEY, G.D. & MOSES, H.L. (1986). Production of transforming growth factors by human colon cancer cell lines. *Cancer Res.*, **46**, 1164.

DERYNCK, R., GOEDDEL, D.V., ULLRICH, A. & 4 others (1987). Synthesis of messenger RNAs for transforming growth factors α and β and the epidermal growth factor receptor by human tumours. *Cancer Res.*, **47**, 707.

DERYNCK, R., ROSENTHAL, A., LINDQUIST, P.B., BRINGMAN, T.S. & GOEDDEL, D.V. (1986). Endogenous and heterologous expression of Transforming growth factorα in mammalian cells. *Cold Spring Harbour Symp. Quant. Biol.*, **51**, 649.

HALPER, J. & MOSES, H.L. (1987). Purification and characterization of a novel transforming growth factor. *Cancer Res.*, **47**, 4552.

KAMATA, N., CHIDA, K., RIKIMARU, K., HORIKOSHI, M., ENOMOTO, S. & KUROKI, T. (1986). Growth inhibitory effects of epidermal growth factor and overexpression of its receptors on human squamous cell carcinomas in culture. *Cancer Res.*, **46**, 1648.

KUDLOW, J.E., KHOSRAVI, M.J., KOBRIN, M.S. & MAK, W.M. (1984). Inability of anti-epidermal growth factor receptor monoclonal antibody to block 'autocrine' growth stimulation in transforming growth factor-secreting melanoma cells. *J. Biol. Chem.*, **259**, 11895.

LEE, K., TANAKA, M., HATANAKA, M. & KUZE, F. (1987). Reciprocal effects of epidermal growth factor and transforming growth factorβ on the anchorage-dependent and -independent growth of A431 epidermoid carcinoma cells. *Exp. Cell Res.*, **173**, 156.

LOWRY, O.H., ROSEBROUGH, N.J., FARR, A.L. & RANDALL, R.J. (1951). Protein measurement with the folin phenol reagent. *J. Biol. Chem.*, **193**, 265.

LYONS, R.M., KESKI-OJA, J. & MOSES, H.L. (1988). Proteolytic activation of latent transforming growth factor-β from fibroblast-conditioned medium. *J. Cell Biol.*, **106**, 1659.

MARQUARDT, H., HUNKAPILLER, M.W., HOOD, L.E. & 4 others (1983). Transforming growth factors produced by retrovirus-transformed rodent fibroblasts and human melanoma cells: Amino acid sequence homology with epidermal growth factor. *Proc. Natl Acad. Sci. USA*, **80**, 4684.

MASUI, T., WAKEFIELD, L.M., LECHNER, J.F., LA VECK, M.A., SPORN, M.B. & HARRIS, C.C. (1986). Type beta transforming growth factor is the primary differentiation-inducing serum factor for normal human bronchial epithelial cells. *Proc. Natl Acad. Sci. USA*, **83**, 2438.

MIYAZONO, K., HELLMAN, U., WERNSTEDT, C. & HELDIN, C. (1988). Latent high molecular weight complex of transforming growth factorβ1. *J. Biol. Chem.*, **263**, 6407.

MOSES, H.L., COFFEY, R.J., LEOF, E.B., LYONS, R.M. & KESKI-OJA, J. (1987). Transforming growth factorβ regulation of cell proliferation. *J. Cell. Physiol. Suppl.*, **5**, 1.

OZAWA, S., UEDA, M., ANDO, N., ABE, O. & SHIMIZU, N. (1987). High incidence of EGF receptor hyper-production in esophageal squamous-cell carcinomas. *Int. J. Cancer*, **39**, 333.

REISS, M. & SARTORELLI, A.C. (1987). Regulation of growth and differentiation of human keratinocytes by type β transforming growth factor and epidermal growth factor. *Cancer Res.*, **47**, 6705.

RHEINWALD, J.G. & BECKETT, M.A. (1981). Tumorigenic keratinocyte lines requiring anchorage and fibroblast support cultured from human squamous carcinomas. *Cancer Res.*, **41**, 1657.

RIZZINO, A., RUFF, E. & RIZZINO, H. (1986). Induction and modulation of anchorage-independent growth by platelet-derived growth factor, fibroblast growth factor and transforming growth factorβ. *Cancer Res.*, **46**, 2816.

ROBERTS, A.B., ANZANO, M.A., WAKEFIELD, L.M., ROCHE, N.S., STERN, D.F. & SPORN, M.B. (1985). Type β transforming growth factor: a bifunctional regulator of cellular growth. *Proc. Natl Acad. Sci. USA*, **82**, 119.

SHIPLEY, G.D., PITTELKOW, M.R., WILLE, J.J., SCOTT, R.E. & MOSES, H.L. (1986). Reversible inhibition of normal pro-keratinocyte proliferation by type β transforming growth factor/growth inhibitor in serum-free medium. *Cancer Res.*, **46**, 2068.

SPORN, M.B. & ROBERTS, A.B. (1985). Autocrine growth factors and cancer. *Nature*, **313**, 745.

TODARO, G.J., FRYLING, C. & DE LARCO, J.E. (1980). Transforming growth factors produced by certain human tumour cells: polypeptides that interact with epidermal growth factor receptors. *Proc. Natl Acad. Sci. USA*, **77**, 5258.

VEALE, R.B. & THORNLEY, A.L. (1989). Increased single class low-affinity EGF receptors expressed by human oesophageal squamous carcinoma cell lines. *S. Afr. J. Sci.*, **85**, 375.

WAKEFIELD, L.M., SMITH, D.M., MASUI, T., HARRIS, C.C. & SPORN, M.B. (1987). Distribution and modulation of the cellular receptor for transforming growth factor β. *J. Cell Biol.*, **105**, 965.